# Cardiac surgery-associated acute kidney injury

**DOI:** 10.1016/j.bjae.2025.11.001

**Published:** 2025-12-08

**Authors:** M.J.P. Oosterom-Eijmael, H. Hermanns, Y.R. Lankadeva, A.H. Hulst

**Affiliations:** 1 Amsterdam University Medical Center, Amsterdam, The Netherlands; 2 Amsterdam Cardiovascular Sciences Research Institute, Amsterdam, The Netherlands; 3 The University of Melbourne, Melbourne, VIC, Australia; 4 Department of Anaesthesia, Austin Hospital, VIC, Australia; 5 Amsterdam Gastroenterology Endocrinology Metabolism Research Institute, Amsterdam, The Netherlands

**Keywords:** acute kidney injury, cardiac surgical procedures, postoperative complications

**Learning objectives**

By reading this article, you should be able to:

• Diagnose cardiac surgery-associated acute kidney injury (CSA-AKI) and identify the biomarkers currently available and under investigation.
• Explain the underlying pathophysiological mechanisms of CSA-AKI.
• Discuss the risk factors that increase the likelihood of CSA-AKI.
• Describe the available preventive strategies.

**Key points**

• CSA-AKI is a common complication and associated with adverse outcomes.
• Key factors in the pathophysiology of CSA-AKI are hypoperfusion, hypoxia, inflammation, oxidative stress and neurohumoral activation.
• The Kidney Disease: Improving Global Outcomes (KDIGO) criteria are commonly used to diagnose and stage CSA-AKI.
• Factors that reduce oxygen delivery to the kidneys or diminish functional renal reserve increase the risk of CSA-AKI.

Cardiac surgery-associated acute kidney injury (CSA-AKI) describes a decline in kidney function after cardiac surgery. Cardiac surgery-associated AKI is the second leading cause of AKI in patients in the ICU (sepsis is the leading cause).^1^ The reported incidence varies from 5% to 40% depending on the type of cardiac surgery, the study group and the definition of AKI.^1^, ^2^, ^3^ Among cardiac procedures, the lowest incidence is after isolated coronary artery bypass graft (CABG) surgery, with progressively increased rates after valvular surgery, aortic surgery and heart transplantation.^3^ Cardiac surgery-associated AKI is associated with short-term complications, including infections, prolonged ICU stay and an increased risk of cardiovascular complications, such as myocardial infarction, stroke and heart failure.^1^

Acute kidney injury is not only associated with chronic kidney disease (CKD) but may also play a direct causal role in its development.^2^ Patients with CSA-AKI have a five-fold increased risk of developing CKD.^2^ Cardiac surgery-associated AKI is associated with an increased risk of mortality extending up to 10 yrs after cardiac surgery.^1^^,^^2^

As CSA-AKI is common and associated with adverse outcomes, clinicians should be able to identify patients at risk, make an early diagnosis and know what strategies to consider for prevention and treatment.

An extended reference list is provided in the Supplementary material and can be used for further consultation.

## Pathophysiology

**Fig 1:**
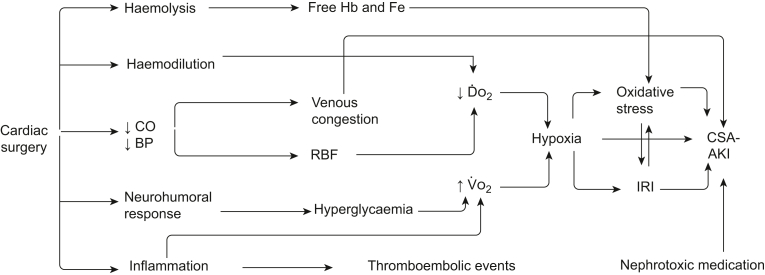
Summary of the pathophysiology of CSA-AKI. CO, cardiac output; CPB, cardiopulmonary bypass; CSA-AKI, cardiac surgery-associated acute kidney injury; Ḋo_2_, oxygen delivery; Fe, iron; Hb, haemoglobin; IRI, ischaemia–reperfusion injury; RBF, renal blood flow; $\dot{\text{V}}$o_2_, oxygen consumption.

The pathophysiology of CSA-AKI is complex, involving multiple interacting pathways and self-perpetuating cycles. Nonetheless, these mechanisms converge into a few key central mechanisms leading to AKI: hypoperfusion and hypoxia, resulting in ischaemia–reperfusion injury, neurohumoral responses, inflammation and oxidative stress (Fig. 1).

### Hypoperfusion

Anaesthesia, cardiopulmonary bypass (CPB), aortic cross-clamping, cardioplegia and manipulation of the heart can all contribute to compromised cardiovascular function.^2^ Reduced cardiac output and hypotension lead to reduced renal blood flow (RBF), particularly in the renal medulla.^4^

The kidneys have two autoregulatory mechanisms that influence RBF by regulating the tone of the afferent arteriole: the myogenic response and the tubuloglomerular feedback.^4^^,^^5^ The myogenic response is rapid and adjusts afferent arteriolar tone in response to the pressure inside the arteriole through changes in the vascular smooth muscle cells. The myogenic response acts to maintain stable RBF. Tubuloglomerular feedback is a slower response mediated by the macula densa, with the aim of regulating glomerular filtration rate (GFR) rather than RBF. Glomerular filtration rate is maintained by adjusting afferent arteriolar tone. For example, an increase in sodium delivery to the distal convoluted tubule of the nephron, detected at the macula densa, leads to vasoconstriction of the afferent arteriole.^4^

Renal autoregulation is impaired during cardiac surgery because systemic arterial blood pressure decreases below the kidney’s autoregulatory threshold (mean arterial pressure approximately 80 mmHg), and because of factors such as hypothermia, haemodilution and non-pulsatile CPB flow.^2^^,^^4^ The kidney is inherently vulnerable to hypoperfusion because of the two capillary networks positioned in series, which require a higher driving pressure compared with other organs where capillary beds are in parallel.^6^ As a result, any upstream vasoconstriction or reduction in GFR can lead to decreased renal medullary perfusion, which limits the ability of the medulla to autoregulate effectively. Furthermore, the body prioritises adequate blood flow to other organs (e.g. heart, brain spinal cord), over the kidneys.^6^ Consequently, blood is redistributed away from the kidneys during CPB.^7^

In addition to these intrinsic mechanisms, renal perfusion is also influenced by the vasoactive neurohumoral response (discussed below) and vasoactive mediators such as nitric oxide (NO), which is a potent systemic vasodilator. The renal medulla relies more on NO for vasodilation and maintenance of microcirculatory perfusion compared with the cortex.^5^ Cardiac surgery-induced haemolysis lowers NO concentration, limiting its protective vascular effects.^5^

Kidney injury is caused not only by arterial hypoperfusion but also by venous congestion.^2^ Increased venous pressure in the kidney is associated with a proportional decrease in GFR and sodium excretion.^2^

### Hypoxia

**Fig 2:**
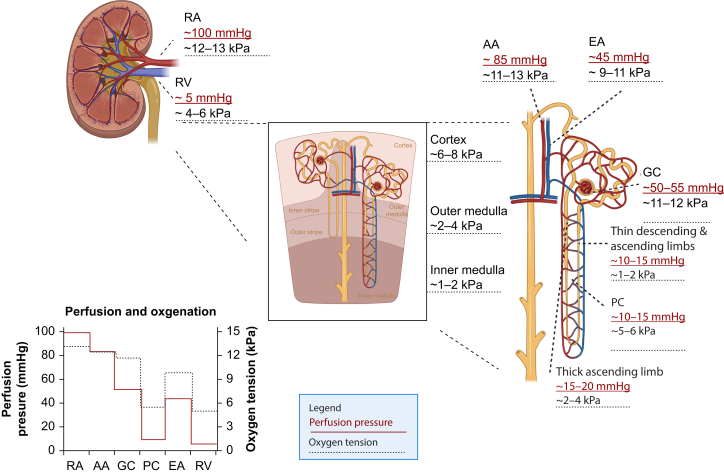
Overview of the perfusion pressure and oxygen tension in different parts of the kidney. Perfusion pressure is shown in red and oxygen tension in black. AA, afferent arteriole; EA, efferent arteriole; GC, glomerular capillaries; PC, peritubular capillaries; RA, renal artery; RV, renal vein.

The kidneys have a high oxygen demand, making them particularly vulnerable to ischaemia.^5^ Moreover, the available oxygen is not evenly distributed throughout the kidney; the oxygen tension in the cortex is around 50 mmHg, compared with 10–30 mmHg in the medulla (Fig. 2).^2^^,^^5^ The active reabsorption of sodium in the proximal tubules and the thick ascending limb of the loop of Henle is responsible for approximately 80% of the kidney’s total oxygen consumption.^5^ The amount of oxygen needed for sodium reabsorption differs throughout the kidney but is higher in the proximal tubule than the loop of Henle.^5^

There is a complex balance between oxygen supply and demand in the kidneys. The kidneys receive one-fifth of the total cardiac output but consume only 5% of the available oxygen in the body.^5^ The high RBF (relative to oxygen consumption) is necessary for the filtration and excretory functions of the kidney. Shunting of oxygen occurs between adjacent arteries and veins in the renal cortex. Oxygen shunting in the cortex may protect against medullary hyperoxia but limits oxygen delivery to the outer medulla, predisposing to hypoxia during periods of reduced RBF.^2^^,^^5^ During cardiac surgery, renal oxygen supply is decreased because of reduced RBF and haemodilution. Haemodilution, an unavoidable consequence of CPB requiring priming circuits, decreases the amount of oxygen per litre of blood.^3^ The combined effects of reduced RBF and haemodilution during cardiac surgery result in a roughly 20% decrease in oxygen delivery to the kidneys.^2^

In addition to reduced RBF, haemodilution and oxygen shunting, stress-induced hyperglycaemia contributes to perioperative kidney hypoxia. Hyperglycaemia, caused by drugs (e.g. corticosteroids) and the proinflammatory state, leads to insulin resistance.^8^ Hyperglycaemia increases oxygen consumption because of increased reabsorption of filtered glucose in the proximal tubules, which is cotransported with sodium.^5^

The kidney’s primary compensatory mechanism for oxygen supply–demand mismatch is to increase RBF, which also increases GFR.^5^ However, increased GFR results in increased sodium reabsorption and, consequently, higher oxygen consumption.^5^ When compensation fails, there are two additional mechanisms to reduce renal oxygen consumption. First, the filtration fraction can be altered by changes in the pre- and postglomerular vascular resistance.^5^ Second, metabolic efficiency can be enhanced by shifting sodium reabsorption to nephron segments that rely more on passive transport, and by varying mitochondrial efficiency in adenosine triphosphate production.^5^ Finally, renal oxygen extraction can be increased. During cardiac surgery, oxygen extraction can increase by approximately 35%, by an unknown mechanism.^7^ Hypoxia persists beyond the duration of surgery, with both medullary and cortical hypoxia still present up to 4 weeks after CPB, which may contribute to the development of kidney fibrosis.^9^

### Neurohumoral response

When the arterial baroreceptors in the carotid arteries and aortic arch detect low arterial pressure (e.g. caused by general anaesthesia or during CPB), they activate the sympathetic neurohumoral response.^2^ The efferent nerves in the kidney release noradrenaline (norepinephrine), activating afferent and efferent arteriolar α_1_ and α_2_ adrenoreceptors, leading to vasoconstriction and reduced RBF.^2^^,^^5^ Noradrenaline also stimulates the production of renin, part of the renin–angiotensin–aldosterone system (RAAS).^5^

Angiotensin II is the most potent product of the RAAS.^5^ Activation of renal AT_1_ receptors by angiotensin II causes vasoconstriction of the efferent and (to a lesser extent) the afferent arterioles.^5^ The net effect is to increase GFR while reducing RBF, resulting in a higher filtration fraction and lower oxygen tension in the renal cortex.

### Inflammation

Inflammation is triggered by the release of damage-associated molecular patterns (DAMPs) by injured tissues and the contact of blood with synthetic surfaces. Hypoxia also induces an inflammatory response, with the potential for a self-reinforcing cycle characterised by progressively increasing metabolic activity, and oxygen consumption.^1^ Hypoxia and DAMPs activate the complement pathway and thereby promote further inflammation, for example via the release of cytokines.^1^^,^^3^^,^^10^ The complement system is also activated by exposure of blood to the CPB circuit and by the use of protamine.^10^ Activation of the complement system triggers mast cell degranulation and leukocyte activation, promotes neutrophil chemotaxis and adhesion to the endothelium and leads to the formation of the membrane attack complex.^10^ This, in turn, results in vasodilation, increased capillary permeability and an amplified inflammatory response.^10^

Inflammation is also triggered by reperfusion. As blood flow is restored, hypoperfusion-related damage may worsen because of leukocyte infiltration into injured kidney tissue.^1^ Immune cells release proinflammatory cytokines and reactive oxygen species, initiating a cascade of further tissue damage, in a process known as ischaemia–reperfusion injury.^1^

### Oxidative stress

Oxidative stress develops as a result of interrupted aerobic respiration during hypoxia, leading to the accumulation of the metabolite succinate.^11^ During reperfusion, oxidation of the accumulated succinate generates superoxide, a reactive oxygen species (ROS).^11^ Reactive oxygen species cause mitochondrial damage and trigger the release of DAMPS and other proinflammatory factors, which in turn contribute to further inflammatory injury.^11^

Furthermore, CPB induces haemolysis, resulting in the release of free haemoglobin and iron into the plasma.^1^ Free haemoglobin increases oxidative stress and binds to NO, thereby inhibiting it, causing vasoconstriction and subsequent ischaemia in the renal microcirculation.^5^^,^^12^ Finally, free haemoglobin can also obstruct the tubules by the formation of haem casts.^12^

Consequently, hypoperfusion, hypoxia, neurohumoral activation, inflammation and oxidative stress are central features contributing to the development of CSA-AKI. Hence, these also form potential targets for kidney-protective interventions and offer a framework for anaesthetists to monitor and manage the risk of AKI.

## Risk factors

Risk factors for the development of CSA-AKI can be categorised into patient-specific and surgery-specific. Comorbidities that reduce oxygen delivery to the kidneys (e.g. pulmonary disease or anaemia), pre-existing CKD or diminished functional renal reserve increase the risk of CSA-AKI.^1^ Characteristics associated with CSA-AKI include advanced age, being non-White and preoperative hypertension.^1^ Female sex has long been considered an independent risk factor for CSA-AKI, but this has recently been challenged as women undergoing cardiac surgery are on average older and have more comorbidities than men. In one study, when analyses were corrected for age and medical history, female sex was not associated with a higher risk of CSA-AKI.^13^

Surgical factors are primarily related to the duration and complexity of surgery and to the occurrence of complications. Surgical risk factors include duration of surgery, CPB time and aortic clamp time, hypoperfusion or hypovolaemia during or after surgery, low flow phases during CPB, use of vasoactive drugs, venous congestion, blood transfusion, emergency surgery and the use of an intra-aortic balloon pump.^1^^,^^3^

Numerous predictive models have been developed for CSA-AKI. The three most commonly known are the Cleveland Clinic model, the Mehta score and the Simplified Renal Index (Table 1).^3^ The models include different variables, although four are consistently used: preoperative kidney function, diabetes mellitus, previous cardiac surgery and type of cardiac surgery.^3^ Validation studies have shown no relevant differences between the Cleveland model and Mehta score.^14^ A potential advantage of the Simplified Renal Index is the inclusion of fewer variables. However, this is offset by lower predictive accuracy than other scoring systems.^14^ The Leicester score is the first score to predict any stage of CSA-AKI, so also includes mild (stage 1) AKI.^15^ A Spanish study confirmed that the Leicester score could be used to select high-risk patients for future interventional trials.^15^ Despite the availability of various predictive models, they are rarely used in clinical practice, likely because of the absence of a subsequent intervention.

**Table 1:** Included variables in the different predictive models for cardiac surgery-associated acute kidney injury. COPD, chronic obstructive pulmonary disease; GFR, glomerular filtration rate.

Cleveland Clinic model	Mehta score	Simplified Renal Index	Leicester score
Female sex	Age ≥55 yrs		Age
Preoperative intra-aortic balloon pump	Non-White race	Preoperative intra-aortic balloon pump	BMI
Insulin-dependent diabetes	Diabetes treated with oral medication	Diabetes requiring medication	Diabetes mellitus
Congestive heart failure	Insulin-dependent diabetes		Left ventricular function
COPD	COPD		Smoking
Preoperative creatinine	Preoperative creatinine	Preoperative GFR	Preoperative GFR
Left ventricular ejection fraction <35%	New York Heart Association class 4 heart failure	Left ventricular ejection fraction <40%	New York Heart Association class
Type of surgery	Type of surgery	Type of surgery	Type of surgery
Emergency surgery	Recent myocardial infarction	Nonelective surgery	Urgency
Previous cardiac surgery	Previous cardiac surgery	Previous cardiac surgery	Time from catheterisation to surgery
	Cardiogenic shock		Hypertension
			Peripheral vascular disease
			Triple vessel coronary disease

## Diagnosis

In the past, several different criteria were used to diagnose CSA-AKI, such as the Acute Kidney Injury Network (AKIN) and the Risk, Injury, Failure, Loss of kidney function and End-stage kidney disease (RIFLE) criteria. Today, the Kidney Disease: Improving Global Outcomes (KDIGO) criteria are the most commonly used; they provide improved sensitivity compared with the AKIN and RIFLE criteria.^1^ The KDIGO criteria classify AKI into stages 1 to 3, depending on serum creatinine concentration and urine output (Table 2).^1^ Although the KDIGO criteria are the currently accepted standard for diagnosing AKI, they have important limitations. Serum creatinine concentration is influenced by muscle mass and dietary protein intake, both of which may vary during the perioperative period.^3^ Furthermore, creatinine concentration does not begin to increase until GFR is <50% of normal.^2^ This delay is further influenced by the renal functional reserve, which allows the kidney to compensate through hyperfiltration.^16^ Cystatin C might be a good alternative to creatinine as a test of kidney function, as it is less dependent on muscle mass and dietary intake and rapidly increases with the onset of AKI.^2^ However, cystatin C is more expensive and less widely available than creatinine.

**Table 2:** Definition of Acute Kidney Injury according to Kidney Disease Improving Global Outcomes (KDIGO) criteria. AKI, acute kidney injury.

AKI stage	Creatinine and urine output definitions		Time frame
1	Increase in serum creatinine	≥26.5 μmol L^−1^ (0.3 mg dL^-1^) OR	Within 48 h
		≥1.5 times baseline	Within 7 days
	Urine output	<0.5 ml kg^−1^ h^−1^	For 6–12 h
2	Increase in serum creatinine	2.0 – 2.9 times baseline	
	Urine output	<0.5 ml kg^−1^ h^−1^	For >12 h
3	Increase in serum creatinine	3.0 times baseline OR	Within 7 days
		≥353.6 μmol L^−1^ (4.0 mg dL^−1^)	
	Start renal replacement therapy		
	Urine output	<0.3 ml kg^−1^ h^−1^ OR	>24 h
		Anuria	>12 h

Urine output as a marker of renal function is also problematic, as it is influenced not only by kidney function but also by the patient’s fluid balance and use of diuretics.^2^ Oliguria loses some of its diagnostic value when clinicians (un)consciously intervene when urine output is reduced.^17^ Urine output is nice example of Goodhart’s Law, ‘When a measure becomes a target, it ceases to be a good measure’.^17^

Much research has attempted to develop new and better biomarkers for AKI (Table 3), with experts recommending the integration of biomarkers into the staging of AKI.^18^ The ideal biomarker would allow for early detection, have good sensitivity for screening and diagnosis, be strongly specific for (a type of) kidney injury, correlate with injury severity, have prognostic or predictive value for AKI or long-term effects and be widely available at minimal cost.

**Table 3:** Summary of four kidney biomarkers. AUC, area under the receiver operating characteristic curve; CKD, chronic kidney disease; DKK3, dickkopf-3; FDA, Food and Drug Administration; IGFBP7, insulin-like growth factors binding protein 7; IRI, ischaemia–reperfusion injury; KIM-1, kidney injury molecule 1; NGAL, neutrophil gelatinase-associated lipoprotein; TIMP-2, tissue inhibitor of metalloproteinase 2.

Biomarker	Measures	Sensitivity (%)	Specificity (%)	Availability	Advantages	Disadvantages
Nephrocheck (TIMP-2∗ IGFBP7)	Cell-cycle arrest proteins	60	88	Available, FDA approved	Early increase after surgery	Not a specific for cause of injury, influenced by inflammation, expensive, inversely correlated with urine output
NGAL	Lipoprotein released by damaged tubular cells	70	85	Urine and plasma assays available	Early increase after surgery	Increased in inflammation and infection, not kidney specific, best in people with normal baseline kidney function
KIM-1	Transmembrane protein upregulated after IRI	74	84	Limited availability, mainly research	Marker of tubular necrosis	Slower increase, lower AUC, expensive, probably only useful in combination panel
DKK3	Protein increased during tubular stress and fibrotic activation	76	79	Not for clinical use, research only	Could potentially identify patients at risk of CKD	Limited validation

Of the numerous biomarkers currently being investigated, tissue inhibitor of metalloproteinase 2 (TIMP-2) and insulin-like growth factors binding protein 7 (IGFBP7) are the first biomarkers available for clinical use and combined under the name ‘NephroCheck’. Both biomarkers are cell-cycle arrest proteins, inducing G1 cell-cycle arrest in response to injury and produced in the proximal tubular cells.^3^^,^^18^ The measurement of TIMP-2 and IGFBP7 in urine is now used to predict CSA-AKI in the early (4 h after cardiac surgery) postoperative period, allowing early initiation of potential interventions to mitigate AKI.^18^ When used at the time of admission to the ICU, the sensitivity and specificity are 0.60 and 0.88, respectively.^19^ However, urine osmolality should be taken into account, as dilute urine samples may yield biomarker concentrations that appear reduced relative to the actual severity of AKI.^19^ Other research found that TIMP-2 and IGFBP7 concentrations are inversely correlated with urine output and that, when corrected for urine output, there was no independent association with AKI.^20^

Neutrophil gelatinase-associated lipoprotein (NGAL) is another biomarker for AKI, which can be measured in serum and urine after secretion by damaged tubular cells.^3^ Concentrations of NGAL increase quickly and peak 6 h after cardiac surgery.^21^ The sensitivity and specificity are approximately 70% and 85%, respectively, with an area under the receiver operating characteristic curve (AUC) of 0.79–0.87.^21^ Disadvantages of NGAL include that it is also secreted by organs other than the kidneys, such as the lungs and liver, leading to increased concentrations in cardiovascular and oncological diseases, and that its diagnostic accuracy is highest in patients with normal kidney function, as NGAL concentrations are also increased in patients with CKD.^21^

Kidney injury molecule 1 (KIM-1), a transmembrane glycoprotein, is a biomarker of the proximal tubule that is upregulated after ischaemia–reperfusion injury and is associated with postoperative AKI.^3^ The diagnostic value of KIM-1 is somewhat lower compared with other biomarkers with a sensitivity and specificity of approximately 74% and 84%, respectively, with an AUC of 0.62.^22^ Disadvantages of KIM-1 include that it requires further validation studies, is expensive and is not widely available; KIM-1 is probably most useful as part of a panel of biomarkers.^23^

Urinary dickkopf-3 (DKK3), a glycoprotein active in Wnt/β-catenin signalling and activated in epithelial cells of the tubules during stress, is another potential biomarker.^24^ In one study, a high preoperative urinary concentration of DKK3 was associated with an increased risk of postoperative AKI, with a sensitivity and specificity of 76% and 79%, respectively, and an AUC of 0.78.^24^ As the Wnt/β-catenin signalling is important in the pathway of kidney fibrosis, DKK3 might identify patients at risk of CKD progression.^25^ Further research is needed to validate this hypothesis.

Recruiting the renal functional reserve is a novel method to assess the reserve capacity of the kidneys, by measuring the increase in GFR induced by i.v. amino acid loading, which acts as a physiological stressor.^26^ Renal functional reserve is significantly lower in patients who develop AKI.^26^

Bladder urinary oxygen tension is under investigation, whereby the oxygen tension of urine in the bladder is used as a surrogate of the oxygen tension in the renal medulla.^12^ Studies have found a strong association between the oxygen tension in the urine and in the renal medulla.^12^ In small clinical studies, patients who develop CSA-AKI have lower intraoperative bladder urine oxygen tensions than those who do not.^27^ The main disadvantage of this technique is its potential unreliability in the presence of anuria, and the possibility of confounding resulting from prolonged ureteric transit time, which increases the opportunity for oxygen diffusion between the urine and the ureteric wall. Larger studies are needed to validate the technique, determine whether urinary oxygen tension could be a useful early predictor of AKI, determine appropriate thresholds and identify the minimal urine output required for reliable measurements.

Finally, renal arterial resistance index (RRI) is a potentially useful tool.^3^ A small study measured intraoperative RRI with transoesophageal echocardiography and observed that the AKI incidence was higher in the group with an increased RRI.^3^ Although these observations are interesting, future studies are required to determine whether this tool offers additional value and is feasible.

## Prevention strategies

Numerous preventative strategies have been investigated for CSA-AKI, with varying degrees of success. Potential strategies are outlined below, grouped according to their targeted mechanisms.

### Hypoperfusion

A large multinational trial compared a continuous infusion amino acids from the start of surgery up to 72 h with placebo and found a reduction in the incidence of AKI in the amino acid group (26.9% *vs* 31.7%; relative risk, 0.85; 95% confidence interval [95% CI], 0.77–0.94; *p* = 0.002; Supplementary material).^1^ The underlying hypothesis was that amino acids reduce the resistance in the afferent arteriole (thereby increasing RBF and GFR) and increase NO activity in the renal cortex and medulla.^1^ However, it is unclear whether the reduced incidence in AKI translates into true kidney-protective effect. As such, future studies are required to validate these findings and confirm that amino acids not only increase GFR but also correspond to a reduction in structural kidney injury.

Several studies have investigated whether a higher arterial pressure target could reduce kidney injury, but have observed no differences in the incidence of AKI (Supplementary material).^2^ However, noradrenaline may be associated with an increased risk of AKI. Vasopressin may be preferable to noradrenaline, as it preferentially constricts the efferent arterioles more than the afferent arterioles, potentially preserving GFR. A study of 300 cardiac surgery patients with vasoplegic shock randomised to vasopressin or noradrenaline showed that the incidence of AKI was lower in the vasopressin group (Supplementary material).^12^ It has also been hypothesised that the kidneys can be prepared for hypoperfusion through a method known as (remote) ischaemic preconditioning, in which brief cycles of ischaemia and reperfusion are applied to protect against subsequent longer periods of ischaemia.^1^^,^^2^ Different techniques of ischaemic preconditioning have been tried, but two large randomised trials have shown no beneficial effects on the incidence of AKI.^12^

Another strategy to improve renal perfusion is the use of NO. Several small studies have demonstrated a reduction in the incidence of AKI after supplementation with NO via the CPB circuit.^12^ However, one study reported increased kidney injury biomarkers in the treatment group, raising concerns about subclinical damage.^28^

Temperature also influences RBF, and the optimal temperature during and after CPB remains under investigation. The Society of Cardiovascular Anaesthesiologists (SCA) advises against rapid rewarming (not exceeding 0.5°C min^−1^) and highlights the importance of avoiding hyperthermia, as arterial blood exiting the CPB circuit at temperatures above 37°C has been associated with an increased risk of kidney injury.^12^

In light of the physiological changes described above and the risks associated with on-pump cardiac surgery, off-pump surgery might be beneficial. Two meta-analyses indeed showed that off-pump surgery reduced the incidence of CSA-AKI (odds ratio, 0.60; 95% CI, 0.43–0.84; *p* = 0.003; and risk ratio, 0.87; 95% CI, 0.77–0.98).^29^^,^^30^ Although off-pump surgery may reduce the incidence of CSA-AKI, its applicability remains limited by surgeon and institutional practices, patient factors and the surgical procedure.

### Hypoxia

Both preoperative anaemia and perioperative blood transfusions are associated with an increased risk of AKI.^3^ Anaemia should be screened for and treated before elective surgery, primarily through iron supplementation and, in selected patients, erythropoietin.^12^

A pilot randomised trial found that the perioperative use of sodium-glucose transporter-2 (SGLT2) inhibitors reduced the incidence of CSA-AKI compared with controls (20% *vs* 66.7%; risk difference, 46.7%; 95% CI, −69.7 to −23.6; *p* < 0.001).^31^ The hypothesised protective mechanism is reduced renal oxygen consumption with SGLT2 inhibitors.^31^ Two large randomised controlled trials are currently underway, which will provide further insight into whether SGLT2 inhibitors reduce the incidence of CSA-AKI.^32^^,^^33^

Previously, it was hypothesised that furosemide, a diuretic that inhibits the Na^+^-2Cl^-^-K^+^ cotransport system in the ascending limb of the loop of Henle, could provide renal protection by reducing the energy and oxygen demands of the kidneys. However, after several negative trials and a Cochrane review, this theory has been abandoned.^34^

Goal-directed perfusion (GDP) may potentially improve renal oxygen supply. GDP involves maintaining specific physiological targets, such as oxygen delivery (Ḋo_2_) and blood pressure, during CPB.^12^ A meta-analysis of a GDP strategy based on Ḋo_2_ thresholds showed that the incidence of AKI was reduced in the intervention group (relative risk, 0.52; 95% CI, 0.38–0.70; *p* < 0.0001).^35^ Although a standardised approach to Ḋo_2_ targets has yet to be established, these encouraging results require further validation.

### Neurohumoral activation, inflammation and oxidative stress

Dexmedetomidine is an α_2_ adrenoreceptor agonist that reduces sympathetic nervous system activity, with the potential to improve kidney perfusion and reduce inflammation.^36^ A 2018 meta-analysis showed that dexmedetomidine was associated with a reduced incidence of CSA-AKI (odds ratio, 0.65; 95% CI, 0.45–0.92; *p* = 0.02).^36^ However, a 2022 clinical practice update by the SCA found that dexmedetomidine was not associated with a reduced risk of CSA-AKI (relative risk, 0.71; 95% CI, 0.41–1.21; *p* = 0.21).^37^ The authors concluded that data on dexmedetomidine are inconsistent and, moreover, that its use is associated with more episodes of hypotension.^37^

Corticosteroids have been investigated for their effects on inflammation related to cardiac surgery in two large randomised trials. In one study, dexamethasone did not reduce kidney failure (1.3% in the dexamethasone group *vs* 1.8% in the placebo group; relative risk, 0.70; 95% CI, 0.44–1.14).^38^ In the other study, methylprednisolone did not reduce the incidence of AKI (40.6% in the methylprednisolone group *vs* 39.2% in the placebo group; relative risk, 1.04; 95% CI, 0.96–1.11).^39^

Sodium bicarbonate can neutralise urinary acidity and thereby reduce the generation of ROS.^40^ Despite this theoretical beneficial effect, a systematic review of five randomised trials showed that sodium bicarbonate did not significantly reduce the incidence of CSA-AKI, the need for renal replacement therapy, ICU length of stay or mortality.^40^

As an antioxidant, vitamin C could theoretically improve RBF and reduce the incidence of CSA-AKI.^41^ However, in a randomised trial, vitamin C (4 g intraoperatively followed by 1 g 8 hourly for 5 days) was not associated with a reduced incidence of CSA-AKI compared with controls.^41^ Some investigators argue that a higher dosage of vitamin C may be necessary to demonstrate its benefits, as suggested by a pilot study in which a high dose increased urine output and reduced vasopressor requirements.^42^ To date, such a high dose has not been investigated in the context of cardiac surgery.

Removing cytokines via extracorporeal blood filtration during CPB may also reduce inflammation. This technique has been investigated mostly to reduce organ dysfunction in patients with sepsis, but a trial in patients undergoing cardiac surgery showed a reduction in the proportion of patients with AKI (28.4% *vs* 39.7% in the control group; *p* = 0.03).^43^

### Recommendations

Many interventions aimed at preventing AKI have proved ineffective. New strategies are currently under investigation, but require further validation before they can be incorporated into clinical guidelines. A recent study demonstrated that variations in surgical practices at both the hospital and clinician level are associated with an increased risk of postoperative AKI, highlighting the need for standardised protocols.^44^ Until new evidence emerges, the current KDIGO guidelines remain the recommended approach for reducing the risk of AKI. The KDIGO guidelines focus on multiple different pathological mechanisms, including improving haemodynamics and glucose control, decreasing the use of nephrotoxic medication and frequent evaluation of kidney function (Table 4). In a multicentre trial, after the KDIGO bundle of care in high-risk patients resulted in a lower incidence of moderate and severe AKI (14% in the treatment group *vs* 24% in the control group; risk difference, 10%; 95% CI, 0.9–19.1; *p* = 0.034).^45^

**Table 4:** Recommendations of the Kidney Disease: Improving Global Outcomes (KDIGO) bundle of care. ACE inhibitor, angiotensin-converting enzyme inhibitor; AKI, acute kidney injury, ARBs, angiotensin receptor blockers; NSAID, nonsteroidal anti-inflammatory drugs.

Advice	Explanation
Discontinue and avoid nephrotoxic agents	Discontinue ACE inhibitors and ARBs for the first 48 h after surgery and avoid NSAIDs, aminoglycosides, contrast media
Ensure volume status and perfusion pressure	Use advanced haemodynamic monitoring for optimisation of the volume status and haemodynamic variables (e.g. mean arterial pressure target ≥65 mmHg)
Close monitoring of serum creatinine and urine output	Diagnose AKI early
Avoid hyperglycaemia	Targeting plasma glucose 110–149 mg dl^−1^ (6.1–8.3 mmol l^−1^) with insulin therapy if required

## Conclusions

Acute kidney injury is common after cardiac surgery and is associated with an increased long-term risk of both CKD and death. The pathophysiology is complex and interacting factors included hypoperfusion, hypoxia, neurohumoral responses, inflammation and oxidative stress. Although interventions such as amino acids and SGLT2 inhibitors show promise, to date, there is no simple preventive strategy for CSA-AKI and the KDIGO bundle of care (Table 4) currently remains the recommended approach.

## Declaration of interest

AHH is BJA Education Board Member. All other authors declare that they have no conflict of interest.

## Funding

Y.R.L. was supported by a National Health and Medical Research Council Emerging Leader Investigator Grant (GNT2025266) and a National Heart Foundation of Australia Future Leader Fellowship (FLF105666). A.H.H. received funding from the European Union & European Union’s Horizon 2020 research (101024833) and innovation programme under the Marie Skłodowska-Curie grant agreement no. 101024833, and from the Netherlands Organisation for Health Research and Development (Veni-09150162410006).

## MCQs

The associated MCQs (to support CME/CPD activity) are accessible at www.bjaed.org/cme/home for subscribers to *BJA Education*.

## Declaration of interest

A.H.H. is an editor and editorial board member of *BJA Education*. The other authors declare that they have no conflicts of interest.
