# Supplementary material for: Cardiac surgery-associated acute kidney injury
Source: BJA Educ. 2025 Dec 8;26(2):92–100. doi: 10.1016/j.bjae.2025.11.001 (PMC12853332; doi:10.1016/j.bjae.2025.11.001)
Supplement: Multimedia component 1 [file mmc1.docx]

# Supplementary reading: additional references relevant to this article

1. Moll V, Zhao M, Minear S, Swaminathan M, Kurz A, Huang J, et al. Continuous Urine Output-Based Alert Identifies Cardiac Surgery-associated Acute Kidney Injury Earlier Than Serum Creatinine: A Prospective and Retrospective Observational Study. J Cardiothorac Vasc Anesth. 2024;38(10):2238-46.
2. Schurle A, Koyner JL. CSA-AKI: Incidence, Epidemiology, Clinical Outcomes, and Economic Impact. J Clin Med. 2021;10(24).
3. Chawla LS, Eggers PW, Star RA, Kimmel PL. Acute kidney injury and chronic kidney disease as interconnected syndromes. N Engl J Med. 2014;371(1):58-66.
4. Lindhardt RB, Rasmussen SB, Riber LP, Lassen JF, Ravn HB. The Impact of Acute Kidney Injury on Chronic Kidney Disease After Cardiac Surgery: A Systematic Review and Meta-analysis. J Cardiothorac Vasc Anesth. 2024;38(8):1760-8.
5. Iguchi N, Kosaka J, Booth LC, Iguchi Y, Evans RG, Bellomo R, et al. Renal perfusion, oxygenation, and sympathetic nerve activity during volatile or intravenous general anaesthesia in sheep. Br J Anaesth. 2019;122(3):342-9.
6. Lankadeva YR, Cochrane AD, Marino B, Iguchi N, Hood SG, Bellomo R, et al. Strategies that improve renal medullary oxygenation during experimental cardiopulmonary bypass may mitigate postoperative acute kidney injury. Kidney Int. 2019;95(6):1338-46.
7. Chen R, Liu D, Zhao H, Wang X. Renal medullary perfusion differs from that in renal cortex in patients with sepsis associated acute kidney injury and correlates with renal function prognosis: A prospective cohort study. Clin Hemorheol Microcirc. 2024;88(2):181-98.
8. Just A, Arendshorst WJ. Dynamics and contribution of mechanisms mediating renal blood flow autoregulation. Am J Physiol Regul Integr Comp Physiol. 2003;285(3):R619-31.
9. Andersson LG, Bratteby LE, Ekroth R, Hallhagen S, Joachimsson PO, van der Linden J, et al. Renal function during cardiopulmonary bypass: influence of pump flow and systemic blood pressure. Eur J Cardiothorac Surg. 1994;8(11):597-602.
10. Gambardella I, Gaudino M, Ronco C, Lau C, Ivascu N, Girardi LN. Congestive kidney failure in cardiac surgery: the relationship between central venous pressure and acute kidney injury. Interact Cardiovasc Thorac Surg. 2016;23(5):800-5.
11. Mullens W, Abrahams Z, Francis GS, Sokos G, Taylor DO, Starling RC, et al. Importance of venous congestion for worsening of renal function in advanced decompensated heart failure. J Am Coll Cardiol. 2009;53(7):589-96.
12. Evans RG, Smith DW, Lee CJ, Ngo JP, Gardiner BS. What Makes the Kidney Susceptible to Hypoxia? Anat Rec (Hoboken). 2020;303(10):2544-52.
13. Zhao L, Li G, Meng F, Sun Z, Liu J. Cortical and medullary oxygenation evaluation of kidneys with renal artery stenosis by BOLD-MRI. PLoS One. 2022;17(3):e0264630.
14. Billings FTt, Jiang Y, Shaw AD. Renal Oxygen Flux during Cardiopulmonary Bypass; Tubular Damage to Preserve Glomerular Filtration-What's a Kidney to Do? Anesthesiology. 2017;126(2):199-201.
15. O'Connor PM, Anderson WP, Kett MM, Evans RG. Renal preglomerular arterial-venous O2 shunting is a structural anti-oxidant defence mechanism of the renal cortex. Clin Exp Pharmacol Physiol. 2006;33(7):637-41.
16. Evans RG, Ince C, Joles JA, Smith DW, May CN, O'Connor PM, et al. Haemodynamic influences on kidney oxygenation: clinical implications of integrative physiology. Clin Exp Pharmacol Physiol. 2013;40(2):106-22.
17. Ranucci M, Aloisio T, Carboni G, Ballotta A, Pistuddi V, Menicanti L, et al. Acute Kidney Injury and Hemodilution During Cardiopulmonary Bypass: A Changing Scenario. Ann Thorac Surg. 2015;100(1):95-100.
18. Gronda E, Palazzuoli A, Iacoviello M, Benevenuto M, Gabrielli D, Arduini A. Renal Oxygen Demand and Nephron Function: Is Glucose a Friend or Foe? Int J Mol Sci. 2023;24(12).
19. Mangieri A. Renin-angiotensin system blockers in cardiac surgery. J Crit Care. 2015;30(3):613-8.
20. Arima S. Role of angiotensin II and endogenous vasodilators in the control of glomerular hemodynamics. Clin Exp Nephrol. 2003;7(3):172-8.
21. Rabb H, Griffin MD, McKay DB, Swaminathan S, Pickkers P, Rosner MH, et al. Inflammation in AKI: Current Understanding, Key Questions, and Knowledge Gaps. J Am Soc Nephrol. 2016;27(2):371-9.
22. Schaer DJ, Schaer CA, Humar R, Vallelian F, Henderson R, Tanaka KA, et al. Navigating Hemolysis and the Renal Implications of Hemoglobin Toxicity in Cardiac Surgery. Anesthesiology. 2024;141(6):1162-74.
23. Sakura T, Kanazawa T, Shimizu T, Shimizu K, Iwasaki T, Morimatsu H. Association between plasma-free haemoglobin and postoperative acute kidney injury in paediatric cardiac surgery: a prospective observational study. BJA Open. 2024;12:100348.
24. Zhang D, Teng J, Luo Z, Ding X, Jiang W. Risk Factors and Prognosis of Acute Kidney Injury after Cardiac Surgery in Patients with Chronic Kidney Disease. Blood Purif. 2023;52(2):166-73.
25. Jufar AH, Evans RG, May CN, Hood SG, Betrie AH, Trask-Marino A, et al. The effects of recruitment of renal functional reserve on renal cortical and medullary oxygenation in non-anesthetized sheep. Acta Physiol (Oxf). 2023;237(4):e13919.
26. Husain-Syed F, Ferrari F, Sharma A, Danesi TH, Bezerra P, Lopez-Giacoman S, et al. Preoperative Renal Functional Reserve Predicts Risk of Acute Kidney Injury After Cardiac Operation. Ann Thorac Surg. 2018;105(4):1094-101.
27. Cho L, Kibbe MR, Bakaeen F, Aggarwal NR, Davis MB, Karmalou T, et al. Cardiac Surgery in Women in the Current Era: What Are the Gaps in Care? Circulation. 2021;144(14):1172-85.
28. Matyal R, Qureshi NQ, Mufarrih SH, Sharkey A, Bose R, Chu LM, et al. Update: Gender differences in CABG outcomes-Have we bridged the gap? PLoS One. 2021;16(9):e0255170.
29. Birnie K, Verheyden V, Pagano D, Bhabra M, Tilling K, Sterne JA, et al. Predictive models for kidney disease: improving global outcomes (KDIGO) defined acute kidney injury in UK cardiac surgery. Crit Care. 2014;18(6):606.
30. Section 2: AKI Definition. Kidney Int Suppl (2011). 2012;2(1):19-36.
31. Yu Y, Li C, Zhu S, Jin L, Hu Y, Ling X, et al. Diagnosis, pathophysiology and preventive strategies for cardiac surgery-associated acute kidney injury: a narrative review. Eur J Med Res. 2023;28(1):45.
32. Vijayan A, Faubel S, Askenazi DJ, Cerda J, Fissell WH, Heung M, et al. Clinical Use of the Urine Biomarker [TIMP-2] x [IGFBP7] for Acute Kidney Injury Risk Assessment. Am J Kidney Dis. 2016;68(1):19-28.
33. Leng J, Li L, Tu H, Luo Y, Cao Z, Zhou K, et al. Mechanism and clinical role of TIMP-2 and IGFBP-7 in cardiac surgery-associated acute kidney injury: A review. Medicine (Baltimore). 2024;103(21):e38124.
34. Meersch M, Schmidt C, Hoffmeier A, Van Aken H, Wempe C, Gerss J, et al. Prevention of cardiac surgery-associated AKI by implementing the KDIGO guidelines in high risk patients identified by biomarkers: the PrevAKI randomized controlled trial. Intensive Care Med. 2017;43(11):1551-61.
35. Romejko K, Markowska M, Niemczyk S. The Review of Current Knowledge on Neutrophil Gelatinase-Associated Lipocalin (NGAL). Int J Mol Sci. 2023;24(13).
36. Silverton NA, Hall IE, Melendez NP, Harris B, Harley JS, Parry SR, et al. Intraoperative Urinary Biomarkers and Acute Kidney Injury After Cardiac Surgery. J Cardiothorac Vasc Anesth. 2021;35(6):1691-700.
37. Lankadeva YR, Kosaka J, Evans RG, Bailey SR, Bellomo R, May CN. Intrarenal and urinary oxygenation during norepinephrine resuscitation in ovine septic acute kidney injury. Kidney Int. 2016;90(1):100-8.
38. Zhu MZL, Martin A, Cochrane AD, Smith JA, Thrift AG, Harrop GK, et al. Urinary hypoxia: an intraoperative marker of risk of cardiac surgery-associated acute kidney injury. Nephrol Dial Transplant. 2018;33(12):2191-201.
39. Wang JY, Song QL, Wang YL, Jiang ZM. Urinary oxygen tension and its role in predicting acute kidney injury: A narrative review. J Clin Anesth. 2024;93:111359.
40. Ngo JP, Lankadeva YR, Zhu MZL, Martin A, Kanki M, Cochrane AD, et al. Factors that confound the prediction of renal medullary oxygenation and risk of acute kidney injury from measurement of bladder urine oxygen tension. Acta Physiol (Oxf). 2019;227(1):e13294.
41. Hertzberg D, Ceder SL, Sartipy U, Lund K, Holzmann MJ. Preoperative Renal Resistive Index Predicts Risk of Acute Kidney Injury in Patients Undergoing Cardiac Surgery. J Cardiothorac Vasc Anesth. 2017;31(3):847-52.
42. Kajal K, Chauhan R, Negi SL, Gourav KP, Panda P, Mahajan S, et al. Intraoperative evaluation of renal resistive index with transesophageal echocardiography for the assessment of acute renal injury in patients undergoing coronary artery bypass grafting surgery: A prospective observational study. Ann Card Anaesth. 2022;25(2):158-63.
43. Landoni G, Monaco F, Ti LK, Baiardo Redaelli M, Bradic N, Comis M, et al. A Randomized Trial of Intravenous Amino Acids for Kidney Protection. N Engl J Med. 2024;391(8):687-98.
44. Kotani Y, Baiardo Redaelli M, Pruna A, Losiggio R, Cocozza S, Ti LK, et al. Intravenous amino acid for kidney protection: current understanding and future perspectives. Clin Kidney J. 2025;18(2):sfae409.
45. Ostermann M, Shaw AD. Amino Acid Infusion to Protect Kidney Function after Cardiac Surgery. N Engl J Med. 2024;391(8):759-60.
46. Kandler K, Nilsson JC, Oturai P, Jensen ME, Moller CH, Clemmesen JO, et al. Higher arterial pressure during cardiopulmonary bypass may not reduce the risk of acute kidney injury. J Cardiothorac Surg. 2019;14(1):107.
47. Vedel AG, Holmgaard F, Rasmussen LS, Langkilde A, Paulson OB, Lange T, et al. High-Target Versus Low-Target Blood Pressure Management During Cardiopulmonary Bypass to Prevent Cerebral Injury in Cardiac Surgery Patients: A Randomized Controlled Trial. Circulation. 2018;137(17):1770-80.
48. Azau A, Markowicz P, Corbeau JJ, Cottineau C, Moreau X, Baufreton C, et al. Increasing mean arterial pressure during cardiac surgery does not reduce the rate of postoperative acute kidney injury. Perfusion. 2014;29(6):496-504.
49. Hajjar LA, Vincent JL, Barbosa Gomes Galas FR*, et al.* Vasopressin versus Norepinephrine in Patients with Vasoplegic Shock after Cardiac Surgery: The VANCS Randomized Controlled Trial. Anesthesiology 2017; **126**: 85-93.
50. Zarbock A, Schmidt C, Van Aken H, Wempe C, Martens S, Zahn PK, et al. Effect of remote ischemic preconditioning on kidney injury among high-risk patients undergoing cardiac surgery: a randomized clinical trial. JAMA. 2015;313(21):2133-41.
51. Martin-Fernandez M, Casanova AG, Jorge-Monjas P, Morales AI, Tamayo E, Lopez Hernandez FJ. A wide scope, pan-comparative, systematic meta-analysis of the efficacy of prophylactic strategies for cardiac surgery-associated acute kidney injury. Biomed Pharmacother. 2024;178:117152.
52. Hausenloy DJ, Candilio L, Evans R, Ariti C, Jenkins DP, Kolvekar S, et al. Remote Ischemic Preconditioning and Outcomes of Cardiac Surgery. N Engl J Med. 2015;373(15):1408-17.
53. Meybohm P, Bein B, Brosteanu O, Cremer J, Gruenewald M, Stoppe C, et al. A Multicenter Trial of Remote Ischemic Preconditioning for Heart Surgery. N Engl J Med. 2015;373(15):1397-407.
54. Kamenshchikov NO, Tyo MA, Berra L, Kravchenko IV, Kozlov BN, Gusakova AM, et al. Perioperative Nitric Oxide Conditioning Reduces Acute Kidney Injury in Cardiac Surgery Patients with Chronic Kidney Disease (the DEFENDER Trial): A Randomized Controlled Trial. Anesthesiology. 2025.
55. Engelman R, Baker RA, Likosky DS, Grigore A, Dickinson TA, Shore-Lesserson L, et al. The Society of Thoracic Surgeons, The Society of Cardiovascular Anesthesiologists, and The American Society of ExtraCorporeal Technology: Clinical Practice Guidelines for Cardiopulmonary Bypass--Temperature Management During Cardiopulmonary Bypass. Ann Thorac Surg. 2015;100(2):748-57.
56. Lankadeva YR, May CN, Bellomo R, Evans RG. Role of perioperative hypotension in postoperative acute kidney injury: a narrative review. Br J Anaesth. 2022;128(6):931-48.
57. Guinn NR, Schwartz J, Arora RC, Morton-Bailey V, Aronson S, Brudney CS, et al. Perioperative Quality Initiative and Enhanced Recovery After Surgery-Cardiac Society Consensus Statement on the Management of Preoperative Anemia and Iron Deficiency in Adult Cardiac Surgery Patients. Anesth Analg. 2022;135(3):532-44.
58. Mahesh B, Yim B, Robson D, Pillai R, Ratnatunga C, Pigott D. Does furosemide prevent renal dysfunction in high-risk cardiac surgical patients? Results of a double-blinded prospective randomised trial. Eur J Cardiothorac Surg. 2008;33(3):370-6.
59. Bayat F, Faritous Z, Aghdaei N, Dabbagh A. A study of the efficacy of furosemide as a prophylaxis of acute renal failure in coronary artery bypass grafting patients: A clinical trial. ARYA Atheroscler. 2015;11(3):173-8.
60. Lassnigg A, Donner E, Grubhofer G, Presterl E, Druml W, Hiesmayr M. Lack of renoprotective effects of dopamine and furosemide during cardiac surgery. J Am Soc Nephrol. 2000;11(1):97-104.
61. Lankadeva YR, Peiris RM, Okazaki N, et al. Reversal of the Pathophysiological Responses to Gram-Negative Sepsis by Megadose Vitamin C. Crit Care Med 2021; 49: e179-e90.
62. Kellum JA, Lameire N, Group KAGW. Diagnosis, evaluation, and management of acute kidney injury: a KDIGO summary (Part 1). Crit Care 2013; 17: 204.
